# Expectations, concerns, and needs of patients who start drugs for chronic conditions. A prospective observational study among community pharmacies in Serbia

**DOI:** 10.1080/13814788.2017.1388778

**Published:** 2017-11-22

**Authors:** Katarina M. Vučićević, Branislava R. Miljković, Bojana C. Golubović, Marija N. Jovanović, Sandra D. Vezmar Kovačević, Milica D. Ćulafić, Milena M. Kovačević, Johan J. de Gier

**Affiliations:** ^a^ Department of Pharmacokinetics and Clinical Pharmacy, Faculty of Pharmacy, University of Belgrade Belgrade Serbia; ^b^ Department of Pharmacotherapy and Pharmaceutical Care, University of Groningen Groningen The Netherlands

**Keywords:** Safety, long-term treatment, community pharmacy, patient–pharmacist counselling

## Abstract

**Background:** During the initiation of treatment of a chronic disease, patients may have varying interests, expectations, concerns, and reasons to stop treatment, influencing compliance with prescribed treatment. Thus, healthcare professionals are expected to integrate these needs into medicines management.

**Objectives:** To determine what information is important to patients; assess predictors of patients’ interests, expectations, concerns, reasons to stop therapy; evaluate drug-related problems following initiation of therapy and summarize how pharmacists resolve them during patient–pharmacist counselling.

**Methods:** In 2014, a four-month study was performed in Serbian community pharmacies, as part of the Pharmaceutical Care Quality Indicators Project led by the European Directorate for the Quality of Medicines & Healthcare. Seventy community pharmacists were asked to participate in the study. Pharmacists recruited adult patients who consented to participate in the study and who initiated treatment, lasting at least six months. Patients completed an open-ended questions form. After two-to-four weeks, a patient–pharmacist consultation was performed.

**Results:** Forty-four community pharmacists (response rate 62.9%) sent back the completed forms from 391 patients (response rate 67.1%). The total number of dispensed drugs was 403. In terms of drug safety, 29.4% of patients sought information, 32.5% expressed concerns, and 28.1% of patients cited it as a reason to discontinue treatment. During the first weeks of therapy, 18% of patients experienced practical problems, while 27.3% reported adverse drug reactions.

**Conclusion:** Safety issues are a major focus of patients’ prescribed new medicines for long-term treatment.

KEY MESSAGES

- Patients prescribed new medicines for long-term conditions have interests, expectations, and concerns about adverse reactions.
- During therapy initiation, one-fifth of these patients experienced practical problems and around a quarter of patients reports adverse drug reactions.

## Introduction

Patient-centred services in community pharmacy settings are still developing. However, in different countries, various pharmacists’ activities are implemented, leading to the optimal medication therapy outcomes, efficient management of the resources, safer drug use, improvement in the quality of life, and lower hospital admission rates [1–6].

Introducing new medicines for long-term treatment is a critical moment for patients. Consequently, it is important that healthcare professionals perceive and respond to the needs of patients [7]. According to the previous reports, 30–50% of medicines are not taken as recommended [8–10]. Patients benefit from information and counselling on the disease, medicines as well as treatment outcomes, to anticipate appropriate drug use [10–12]. The ability for a patient to retain information about a new medication can be limited by a patient’s capacity to remember information given during the consultations with the prescribing physician. Pharmacists are well positioned within a healthcare team to ensure that patient needs are met [13,14]. Previous studies have shown that community pharmacists can successfully intervene when a patient is prescribed a new medicine(s) for a chronic condition [4,15,16]. Hence, in more than 90% of community pharmacies in England a basic service is provided to the patients to whom new medicines are prescribed [7,10,17,18].

The Committee of Experts on Quality and Safety Standards in Pharmaceutical Practices and Care coordinated by the European Directorate for the Quality of Medicines & Healthcare (EDQM, Council of Europe) initiated a research programme. A questionnaire was developed regarding delivery of pharmaceutical care service in community pharmacies for patients receiving newly prescribed long-term drugs. The concept of pharmaceutical care focuses on the individual patient’s needs and achieving positive therapy outcomes. These are accomplished by steps that include: establishing pharmacist–patient relationship; collecting relevant information directly from the patient or the medical records; identifying potential/actual drug-related problems; developing and implementing a therapy plan together with a patient that will prevent/resolve drug-related problems; and further monitoring and (if needed) modifying the plan so desired therapy outcomes are achieved. The Pharmaceutical Care Quality Indicators Project (PCQIP) was carried out among European countries in 2013–2014 [1,19,20]. It focused on the previously described four steps. The pharmaceutical care concept is well known in the Serbian pharmaceutical sector due to a high interest in postgraduate education programmes of Pharmaceutical Care at the University of Belgrade—Faculty of Pharmacy during the last decade [21]. However, pharmaceutical care practice is not consistent among different pharmacies, as the national regulatory body does not formally recognize those services.

The aim of this study was to determine what information is relevant to patients receiving new medicines for chronic treatment. Furthermore, to assess predictors of patients’ interests (knowledge), expectations, concerns, reasons to stop therapy, and drug-related problems, following the therapy initiation to improve pharmacists’ counselling.

## Methods

### Study design

This prospective observational study was conducted in the Serbian community pharmacies during a four-month period in 2014 as a part of the PCQIP. Hence, all stages of the research were performed in compliance with EDQM study plans and protocols [1,19,20]. The local ethical committee of the University of Belgrade—Faculty of Pharmacy approved this study (no. 2718/2, 18 December 2013).

### Recruitment of pharmacists and selection of patients

The Pharmaceutical Chamber of Serbia announced the study on their website and in the official journal and performed recruitment of the community pharmacists. Seventy community pharmacists applied to participate in the study. Each pharmacist was asked to complete, sign an agreement form and to recruit 5–10 adult patients. Inclusion criteria were patient’s age 18–65 years, who initiated treatment with drugs for alimentary tract and metabolism, cardiovascular, musculoskeletal, respiratory system, lasting at least six months and not used in the previous period. Exclusion criteria included no direct contact with the patient, physically frail elderly, patients receiving palliative care or with cognitive impairment. Each patient was informed about the purpose of the study before signing the consent form.

### Data collection forms

All documents were originally developed by EDQM in the English language. Translation and validation procedures were applied including the process of forward and backward translations, review of the translated questions and their testing in a smaller group.

Patients, who gave informed consent, were asked to complete ‘My checklist’ self-completion concordance form (SCCF) at home [1,2,20]. This form allowed patients to write down information they needed to know, expectations and concerns they had, possible reasons for therapy discontinuation, observed drug-related problems during the first weeks of drug use or any other issues in relation to newly started medicines.

The patient–pharmacist consultation was appointed after two-to-four weeks, and it was directed to the issues reported in SCCF.

Finally, the pharmacists filled ‘Consultation form for pharmacists,’ where they briefly recorded the feedbacks from patients’ answers in SCCFs and provided an overall evaluation and the consultation outcome [1,2,20].

### Outcomes and statistical analysis

The patients’ responses were summarized into several categories and sub-categories (Table 1). The binary coding system was used for numerical transformation of patients’ answers. Descriptive and statistical analyses using binomial logistic regression were performed with IBM SPSS Statistics 18. Data was analysed as a single cohort and per each group according to the Anatomical Therapeutic Chemical Classification System of newly prescribed drugs. Tested covariates were the number of newly prescribed drugs and pharmacology group/individual drug(s) and a model with a constant was built using the backward Wald method. The results of the analysis were presented as odds ratios (OR) and their 95% confidence intervals (95%CI). Statistical significance was considered at *P* < 0.05.

**Table 1.** Examples of sub-categorizing patients’ answers.

Patient’s response	Sub-category
‘Is this drug harmful if I use it for a long period?’	Side effects
‘Do I have to take medicines every day?’	Regimen
‘I expect that the drug will lower my LDL and total cholesterol level.’	Condition under the control
‘How to use these inhalators properly? My doctor told me, but I'd like to hear it again.’	Drug use
‘It seems that this drug is not helping me.’	Ineffectiveness
‘Is this life-long treatment?’	Duration
‘How to keep the drug properly? I heard that I could keep it in the fridge, is it true?’	Storage

## Results

In total, 44 community pharmacists (response rate 62.9%) sent back completed data forms from 391 patients (patients’ response rate 67.1%). The total number of dispensed drugs of interest was *n* = 403, including cardiovascular (*n* = 247), alimentary tract (*n* = 59), musculoskeletal (*n* = 37), and respiratory drugs (*n* = 60).

### Interests (knowledge)

**Figure 1.**
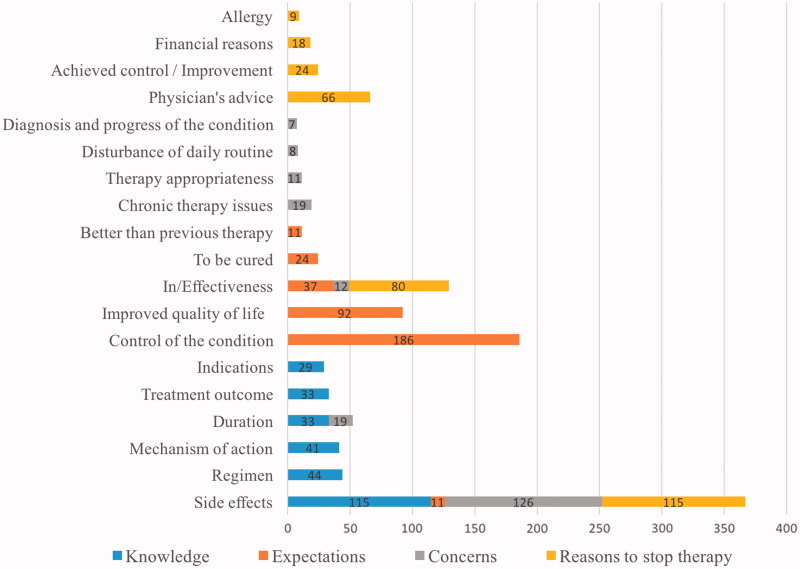
Number of patients’ answers concerning interests (knowledge); expectations; concerns; reasons to stop therapy.

Most patients (84.9%) were interested in receiving drug information beyond what they knew at the moment the drug was dispensed. Mainly, patients were interested in safety profiles (29.4% of patients); dosing regimens and treatment duration (77 patients, 19.7%); and mechanism of action and indication (70 patients, 17.9%) (Figure 1). Results of binary logistic regression indicated that being prescribed ≥3 new drugs was a significant predictor of patients’ seeking additional information about dosing regimen. Prescribed diuretics were a significant additional predictor of the same issue (Table 2). Moreover, patients who were prescribed beta-blockers and statins were 3.5 times more likely to require information about the treatment outcomes (Table 2). However, when respiratory drugs were prescribed, 19% of patients were interested in learning drug use (e.g. practical aspects of the use of the inhalers).

**Table 2.** Statistically significant predictors of patients’ knowledge, expectations, concerns, reasons to stop therapy.

Category	Cohort	Sub-category	Predictor^a^	OR	95%CI
Knowledge	All patients (*n* = 391)	Regimen	≥3 new drugs**	4.31	1.52–12.21
		Treatment outcome	M**	3.63	1.50–8.77
	Patients prescribed C medicines (*n* = 246)	Regimen	≥3 new drugs* Diuretics*	4.28 2.90	1.11–16.58 1.03–8.17
		Treatment outcome	Beta blockers* Statins*	3.35 3.75	1.22–9.16 1.06–13.33
Expectations	All patients (*n* = 391)	To be cured	A** M***	3.80 5.63	1.48–9.73 2.08–15.24
		Quality of life improvement	A** C*** R***	0.25 0.27 4.07	0.09–0.68 0.13–0.57 1.77–9.40
	Patients prescribed C medicines (*n* = 246)	Better control than previous therapy	Calcium channel blockers**	5.72	1.64–19.94
Concerns	All patients (*n* = 391)	Side effects	A* C** M** R**	6.38 11.18 12.86 10.43	1.29–31.62 2.06–60.71 2.22–74.53 1.87–58.28
Reasons to stop treatment	All patients (*n* = 391)	Financial reasons	M** R*	5.59 3.25	1.72–18.10 1.02–10.30
	Patients prescribed C medicines (*n* = 246)	Achieved control	Beta blockers*	4.10	1.26–13.43

^a^ Statistically significant level:

* *P* <0.05,

** *P* <0.01,

*** *P* < 0.001.

OR: odds ratio; 95%CI: 95% confidence interval; A, C, M, R, newly prescribed drugs for alimentary tract and metabolism, cardiovascular, musculoskeletal system, respiratory system, respectively.

### Expectations

Approximately 50% of patients expected that the new drug would control the symptoms of the health problem while 6% of patients expected a permanent solution while almost one quarter anticipated an improvement of the quality of life (Figure 1). By using binary logistic regression we determined that patients, who were prescribed at least one drug for the alimentary or musculoskeletal system, were 3.8–5.6 times more likely to expect a permanent resolution of their health problem (Table 2). Calcium channel blockers were predictive of patients’ expectations that a newly prescribed drug will better control the symptoms than the previous therapy (Table 2).

### Drug-related problems

In this study, 18% of patients experienced problems with administration and dosing regimen during the first weeks of treatment. Similarly, 27.3% of patients experienced adverse drug reactions following drug treatment initiation. The most frequent were gastrointestinal disturbances in 33, central nervous system in 14 patients, followed by cardiovascular adverse effects in 10 patients. Statistical analysis showed that a higher proportion of patients experienced practical problems associated with inhaled corticosteroids when respiratory drugs were prescribed (Table 3). Additional results are provided in Table 3.

**Table 3.** Statistically significant predictors of drug-related problems patients’ reported following the initiation of therapy.

Category	Sample	Sub-category	Predictor*	OR	95%CI
Practical problems	Patients using C class medicines (*n* = 246)	Poor control	≥3 new drugs*	6.77	1.19–38.56
	Patients using R class medicines (*n* = 60)	Administration	Inhaled corticosteroids*	5.00	1.46–17.10
Adverse drug reactions	All patients (*n* = 391)	GIT side effects	R**	0.045	0.006–0.345

^a^ Statistically significant level:

* *P* <0.05,

** *P* <0.01,

*** *P* <0.001.

OR: Odds ratio; 95%CI: 95% confidence interval; A, C, M, R, newly prescribed drugs for alimentary tract and metabolism, cardiovascular, musculoskeletal system, respiratory system, respectively.

### Concerns

In the study, 32.5% of patients were mainly concerned about the drugs’ safety profiles while 9.8% of patients were worried about the duration of therapy. Ineffectiveness was an issue for 3.1% of patients (Figure 1). When respiratory medicines were prescribed, 12% of patients were worried about chronic therapy aspects including forgetfulness, behaviour in case of acute respiratory crisis, etc. Statistical analysis results are given in Table 2.

### Reasons to stop therapy

According to the results, manifested adverse drug reactions (reported by 28.1% of patients), drug’s ineffectiveness (19.5%) and advice given by medical doctors (16.1%) would be potential reasons for treatment termination (Figure 1). If a patient were to experience an improvement in the treated condition, 5.9% patients might stop taking the drug. Treatment discontinuation due to financial reasons was reported by 4.4% of patients, mainly when musculoskeletal and respiratory drugs were prescribed (12.5% and 7.9% of patients, respectively). Accordingly, these patients were approximately five and three times more likely to stop the treatment due to financial issues (Table 2).

### Pharmacist–patient consultation outcome

The patient–pharmacist consultation has been based on patients’ needs, concerns or drug-related problems stated in the SCCF. In total, 59.0% of patients agreed that counselling with pharmacists improved their comprehension of the medication use. Moreover, pharmacists reported that 65.0% of the patients who reported adverse drug reactions were referred to their medical doctor while the pharmacist resolved the remaining drug-related problems (such as a sore throat when corticosteroids inhalers were used, gastrointestinal tract disturbances, etc.) Non-adherence to therapy was recognized in 12.2% of patients, and 45.2% of these patients were referred to their medical doctor, while the remaining patients were educated by pharmacist–patient interaction on the necessity of taking drugs as prescribed. Approximately, 90% of the consultations were self-evaluated as positive by the pharmacist who performed them.

## Discussion

### Main findings

Our results suggest that patients with newly prescribed medicines for treatment of chronic disease are interested and willing to engage in the support offered by the community pharmacists. During the first weeks of therapy initiation, 18% of patients experienced practical problems with drug use and 27% of patients reported adverse drug reactions. Consequently, pharmacists took an active role by resolving 35% of adverse drug reactions reported by patients and 54.8% of adherence issues. Pharmacists referred the remaining patients to their medical doctors due to potential/actual drug-related problems.

### Strengths and limitations

The limitation of the study is the researchers’ interpretations of patients’ answers and coding step as an open-type question form was used. However, two researchers who independently analysed the given answers performed this procedure. There was no statistical calculation of sample size. It is important to highlight that predictors identified in our study (e.g., reason to stop the treatment with musculoskeletal and respiratory drugs due to financial issues) may not completely reflect healthcare systems in other countries where different co-payment schemes exist. Moreover, it was up to the pharmacists’ discretion to provide counselling. It would be useful to include therapy outcomes because of pharmacists’ interventions to evaluate the impact of the pharmaceutical care model at the initiation of the long-term treatment.

### Interpretation and relation to literature

This study is an extension and a supplement to the previously performed studies in The Netherlands and Bulgaria [1]. The distribution of patients’ answers in all categories (Figure 1) is comparable with the results of the studies performed in The Netherlands and Bulgaria [1,2]. In our study, the most common issues raised by patients were the drugs’ safety profiles (Figure 1) as previously reported [1,2,7,12,17,22]. Patients were interested not only in gaining more information but they also had concerns regarding the adverse drug reactions and 28.05% of patients reported that they would discontinue the treatment if side effects would appear.

As previously reported, dosing regimen and outcome counselling are contributing to the high level of adherence during long-term therapies [23,24]. Our results indicate that three or more newly prescribed drugs as well as prescribed diuretics (due to the impact of the time of administration on daily routine or overnight quality of sleep) are significant predictors of patients’ interests in dosing regimen. The results of the study suggest that patients are more likely to require information on the treatment outcomes when prescribed beta-blockers and statins, due to post-myocardial infarction patients’ awareness of the seriousness of their disease and worries about the future cardiovascular events [25] as well as patients prescribed musculoskeletal drugs as disease can limit their everyday routine [26,27].

Patients prescribed with musculoskeletal or respiratory drugs have a higher probability of discontinuing the treatment due to financial issues in Serbia (Table 2). It is interesting to note that for most of these drugs; patients’ cost sharing is on average 5–10 times higher than the basic additional fee for the reimbursement list of medicines. In analysed cohorts, 66% of patients were prescribed drugs requiring an additional fee (up to 35 times greater than a basic additional fee) upon dispensing. As previously observed, this may lead to lower level of adherence and consequently to treatment failure [17,28].

Furthermore, poor control was likely to be reported by patients if three or drugs that are newer were prescribed (with at least one for the cardiovascular system). This poorly controlled drug use may be due to patients’ subjective perception of the not easily recognizable multiple symptoms (e.g., hypertension) [10]. Furthermore, patients on inhaled corticosteroid therapy reported drug administration problems (Table 3). These problems may be partially explained by corticosteroid phobia owing to lack of information, potential side effects that patients are likely to encounter (such as oral candidiasis, sore throat, hoarse voice), and possibly inadequate patients' inhalation techniques, all leading to inappropriate drug use [10,15]. Hence our results identify the patients who may require more comprehensive pharmacist counselling when dispensing drugs in the community pharmacies.

### Implications for clinical practice

Patient-guided counselling allows more supplemental questions by both patient and pharmacist, and a more in-depth recognition of individual patients’ needs, expectations, and concerns when prescribed new medicines for long-term treatments. Information about adverse reactions to newly prescribed drugs for treatment of chronic disease ought to be an integral part of medicine management. Additionally, when patients are prescribed three or more new drugs for treatment of chronic disease, counselling on dosing regimen is warranted. In contrast to multiple drugs regimen, counselling is required whenever respiratory drugs are introduced in the therapy. Frequent patient monitoring would be beneficial in the first few weeks of treatment initiation, as patients may experience drug-related problems. This study can be used to discriminate delicately which patients require individual pharmacists’ attention and which specific aspects should be covered during the critical first weeks when new medicines are prescribed, so that long-term outcomes can be achieved. Hence, these results should be used to improve patient–pharmacists counselling.

## Conclusion

Pharmacists are well positioned within a healthcare system and should take a proactive role in the care of patients with newly prescribed drugs for treatment of chronic disease, aiming to ensure that patients’ expectations and needs are met, concerns are minimal, that patients will adhere to the prescribed treatment, and recognize/resolve drug-related problems during the therapy initiation.
